# Linking Macroinvertebrates and Physicochemical Parameters for Water Quality Assessment in the Lower Basin of the Volta River in Ghana

**DOI:** 10.1007/s00267-021-01535-1

**Published:** 2021-09-16

**Authors:** Michael Onwona Kwakye, Feng-Jiao Peng, Jonathan N. Hogarh, Paul J. Van den Brink

**Affiliations:** 1 Aquatic Ecology and Water Quality Management Group, Wageningen University, P.O. Box 47, 6700 AA Wageningen, The Netherlands; 2 Environmental Protection Agency, P. O. Box M326, Accra, Ghana; 3 Human Biomonitoring Research Unit, Department of Population Health, Luxembourg Institute of Health, 1 A-B rue Thomas Edison, 1445, Strassen, Luxembourg; 4 Department of Environmental Science, College of Science, Kwame Nkrumah University of Science and Technology, PMB University Post Office, Kumasi, Ghana; 5 Wageningen Environmental Research, P.O. Box 47, 6700 AA Wageningen, The Netherlands

**Keywords:** Macroinvertebrates, Physicochemical parameter, Water quality, Ghana

## Abstract

The health of the lower basin of the Volta River in Ghana was evaluated in January–February and May–June 2016 using physicochemical parameters and benthic macroinvertebrates sampled at 10 locations. Selected environmental variables were compared to accepted environmental water quality standard values where applicable. Principal component analysis (PCA) and redundancy analysis (RDA) were used to analyse the association between the benthic macroinvertebrates distribution and physicochemical variables. Pesticide concentrations were generally below the limit of detection 0.01 and 0.005 µg/L for organophosphate/synthetic pyrethroid and organochlorines respectively. Nutrient levels were also generally low; however, significant differences existed between the values of physicochemical parameters at the different sampling sites and seasons (Monte Carlo permutation test; *p* = 0.002), as well as between the abundance of macroinvertebrates at the different sites and seasons (*p* = 0.002). The environmental variables dissolved oxygen (DO), phosphate, pH, substratum (*p* < 0.05), turbidity, conductivity, total dissolved solids, total solids and nitrate (0.05 < *p* < 0.10) significantly explained the variation in macroinvertebrate composition between sampling stations in the Volta River. *Polypedilum fuscipenne*, was positively correlated with turbidity and DO concentrations; *Physa* sp., *Centroptilum* sp., *Centroptiloides* sp., *Phaon iridipennis* and juvenile fish were positively correlated with nitrate concentration and pH and negatively correlated with turbidity and DO. Polluted sites were dominated by the snail *Lymnaea glabra*. This demonstrates that physicochemical parameters and macroinvertebrates could be applied to describe the water quality and improve the biomonitoring for water resources management and the environmental protection in the Lower Volta River.

## Introduction

The Volta River is one of largest river systems in Africa covering an area of ~400,000 km^2^; shared by six riparian states of West Africa and one of the most important river systems in Ghana (Barry et al. 2005). The north-south extent of this transboundary basin stretches from approximately latitude 5^o^ 30′ N in Ghana to 14^o^ 30′ N in Mali, with the widest stretching approximately from longitude 5^o^ 30′ W to 2^o^ 00′ E (Gordon et al. 2013). The lower part of the river basin promotes different uses including agriculture, aquaculture, fishing, water for domestic (drinking) and industrial purposes, water transport, sand mining and industrial activities (e.g. textile works) among others (Andah et al. 2003; Mul et al. 2015). The lower basin also includes two hydroelectric dams (Akosombo and Kpong). The Kpong Dam is 24 km downstream of Akosombo along the river channel and the establishment of the Akosombo dam for example has rendered some of the soils more acidic (Barry et al. 2005). Sedimentation in the river has been reported, resulting from the hydrological alterations of the dams (Boateng et al. 2012; Ly, 1980; Amenuvor et al. 2020). The river also receives domestic wastewater, industrial wastewater, municipal and rural wastes, and other human activities. High levels of organic pollutants may degrade the water quality in receiving waters and threaten the aquatic ecosystems (Asantewaa Owusu et al. 2016; Corcoran et al. 2010; Wang et al. 2013). For example, water may become polluted due to a range of contaminants originating from agricultural activities (Hooda et al. 2000; Karikari, Ansa-Asare (2006); Lovelle, Sullivan (2006)). Indeed, pesticides have been reported to affect water bodies in Ghana (Acquaah (1997); Fianko et al. 2011; Ntow 2001,2005). In addition, the statistics show that the water sources have been, and continue to be, exploited (Asantewaa Owusu et al. 2016). To improve the water resources management and the water quality monitoring for the Volta River system and other water resources, monitoring of physiochemical parameters and aquatic macroinvertebrates have been applied (Baa-Poku et al. 2013; Thorne et al. 2000; Thorne and Williams 1997). In Ghana, however, the application of these monitoring tools to evaluate the relationships between the community composition of benthic macroinvertebrates, physicochemical variables and pesticides for the water quality evaluation is rather limited and in its early stages.

Benthic macroinvertebrates are a ubiquitous and diverse group of species that react strongly and often predictably to human influences in aquatic ecosystems. In addition some are sedentary; therefore, body burdens reflect local conditions, allowing detection of a variety of perturbations in a range of aquatic habitats (Rosenberg and Resh 1993). Benthic macroinvertebrates are an important and integral part of many aquatic ecosystems and any negative effects caused by pollution in the community structure can in turn affect higher trophic levels like fish and birds. Further, benthic invertebrates have the ability to clean waterways as they utilize the organic and detritus matter (Sharma and Chowdhary, 2011). Macroinvertebrate populations in streams and rivers can assist in the assessment of the overall health of the system (e.g. Carlisle et al. 2007).

The overall objectives of the research were to: (1) evaluate the values of the physicochemical parameters and pesticides and benthic macroinvertebrate richness and composition in the Lower Volta River system, and (2) examine the relationships between the environmental variables and the macroinvertebrate community to determine their response to the water quality parameters in the Volta River.

## Materials and Methods

### Study area

There are two distinct types of savannah in the basin: woodland savannah and grassy savannah. The woodland savannah, mostly found in the southern parts of the basin, is densely wooded with tall to medium tall grasses (Mul et al. 2015). The climate of the Volta Basin is dominated by the rain-bearing south westerly tropical maritime air mass and the dry, north easterly tropical continental air mass (Dickson and Benneh 1988). Normally, there is a bimodal rainfall from April to July and from September to November in Southern Ghana. The single wet season is from May to October in Northern Ghana, which is followed by dry season (Harmatan). The wettest area in Ghana is the extreme southwest where annual rainfall is about 2000 mm; the annual rainfall generally decreases from south to north. The country has a high temperature with the average annual temperature ranging between 24 °C and 30 °C (GEPA Ghana Environmental Protection Agency (2011)). In the coastal area of Ghana the relative annual humidity is 95–100% in the morning and about 75% in the afternoon. In the north these values can be as low as 20–30% during the Harmatan period and 70–80% during the rainfall period (Andah et al. 2003).

**Fig. 1:**
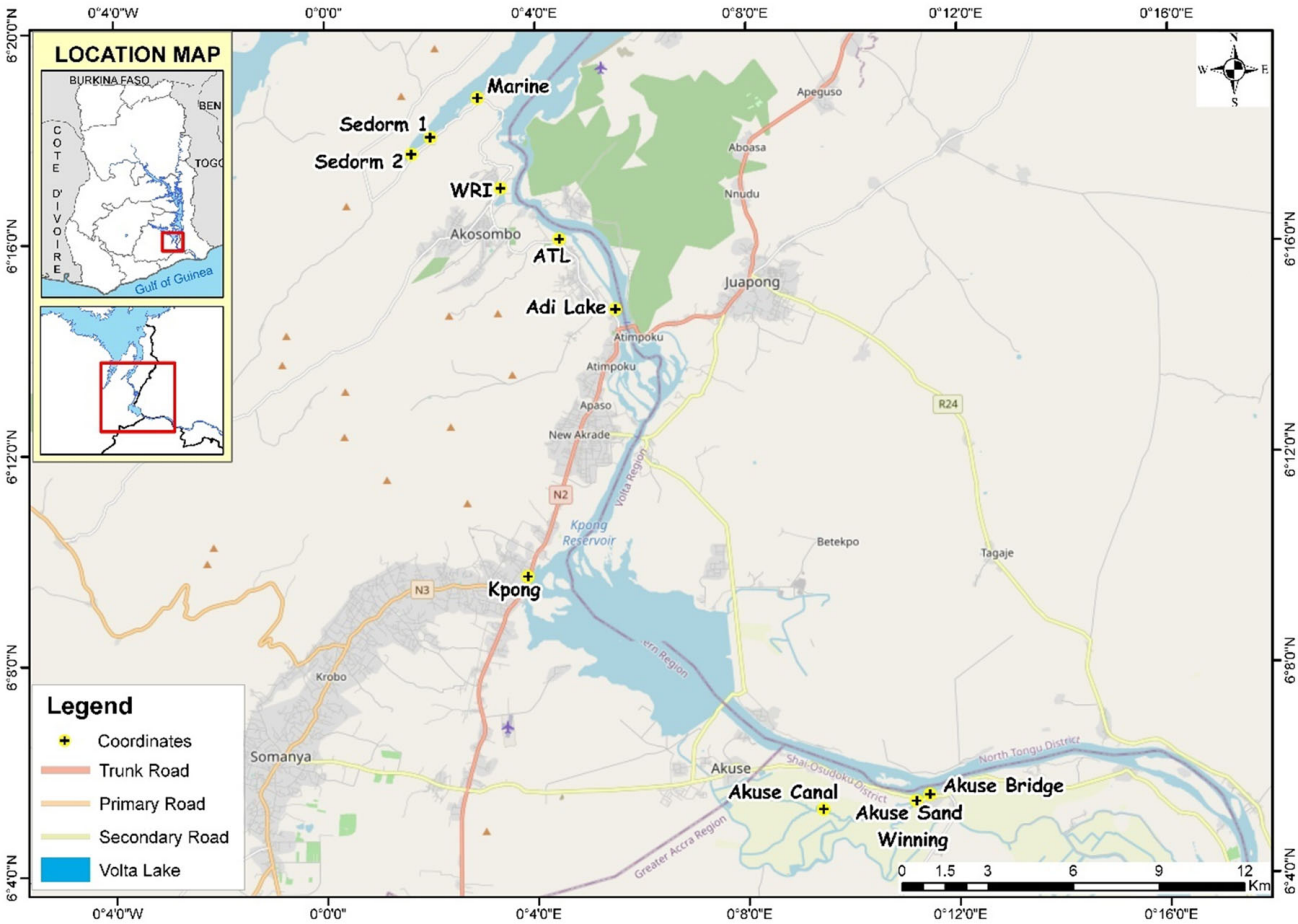
Map showing study area and sampling points

The study area has average rainfall of 1000 mm/year with distinct dry (October–May) and wet (May–October) seasons. Temperatures vary between ~16 and 40 °C depending on season, time of day, and elevation (Bekoe and Logah 2013) and falls within the Dahomeyan system which occurs at the southern part of the main Volta Basin (Fig. 1), and consists of mainly metamorphic rocks, including hornblende and biotite, gneisses, migmatites, granulates, and schist (Barry et al. 2005).

### Site Selection and Sampling

Site selection was based on land use, accessibility and anthropogenic activities using the Rapid Bioassessment Protocol (RBP) for streams and wadable rivers (Barbour et al. 1999). The sampling reach was divided into three areas: the Akosombo hydroelectric Dam (3 stations), in between the Akosombo hydroelectric Dam and Kpong hydroelectric Dam (4 stations) and the Kpong hydroelectric Dam (3 stations) (Fig. 1). Water quality was evaluated in the river by sampling upstream, the hydrologic alteration from the dam, and downstream of the waterways and the differences in macroinvertebrate abundance were used as biological indicator of disturbance (Tables SI 2, 3, and 4). Land uses (Table SI 1) and stations were subjected to non-point influents (i.e. agricultural runoff) and point influents (i.e. fish pond). Also, one site was selected as a reference site where there was no or slight pollution expected (Table SI 1; T6 (Adi Lake)). Each station was sampled three times within a 2-week interval in the dry and wet seasons namely: January–February 2016 and May–June 2016, respectively, for the investigation of physicochemical parameters, pesticide concentrations, and macroinvertebrate abundance. Sampling was mainly confined to a few meters (~4) from the banks of the river courses except on a few occasions where a canoe was used due to unavailability of a conducive bank. Surface water samples were taken from a depth of 20–30 cm. Samples were collected into acid-cleaned high-density 1 L polyethylene bottles. The samples were carried in an ice cooler from the field and stored in a refrigerator at 4 °C before analysis of physicochemical parameters. Water samples were again taken from each of the sites using pre-cleaned sterile glass amber bottles and kept at 4 °C and subsequently used for pesticide analyses.

At each sampling location, a surber sampler (30 × 30 cm and 250 micron mesh) was used for collecting macroinvertebrates based on the RBP as it is suitable to sample different habitats (Lima da Cunha et al. 2019). On each site three replicates were collected and composited as one sample. Benthic macroinvertebrates were preserved in 4% formaldehyde solution. The macroinvertebrates were sorted, identified to the lowest possible taxonomic level (species, genus, or families), and counted under a stereomicroscope.

### Physicochemical Analysis

During sampling, water temperature (°C), pH (−), dissolved oxygen (DO mg/L), total dissolved solids (TDS, mg/L), turbidity (NTU), and electrical conductivity (µs/cm) were measured on site using portable equipment (Horiba U*-*50 Series multi-parameter water quality meter). Total solids (TS) was determined by Gravimetric Method (APHA American Public Health Association (1998)). 10 ml of the water samples were transferred into a pre-weighed evaporating dish which was then dried in an oven at a temperature of 103–105 °C for 2 and half hours. The dish was transferred into a desiccator and allowed to adjust to room temperature and was weighed. The TS was represented by the increase in the weight of the evaporating dish. The total suspended solids were easily obtained by simple calculation, i.e. total suspended solids = total solids − TDS. Biological oxygen demand (BOD) was determined according to standard methods for the examination of water and wastewater (APHA American Public Health Association (1998)).

Orthophosphate (PO_4_ –P) was determined using ammonium molybdate and ascorbic acid method (Mackereth et al. 1978), ammonia-nitrogen (NH_4_ –N) by the indophenol blue method (Franson 1989), nitrate-nitrogen (NO_3_ –N) by hydrazine reduction followed by diazotizing to form an azodye which was measured colorimetrically and nitrite-nitrogen (NO_2_ –N) was determined by N-(1-2 naphthyl) ethylene di amine di -hydrochloride method. (APHA American Public Health Association (1998)). All reagents used were of analytical grade, equipment was calibrated before measurement and replicate analyses were carried out for each determination to ascertain reproducibility and quality assurance.

### Pesticide Extraction and Analysis

The following pesticides were chosen as target compounds based on information of previous and current pesticide use: lindane, delta-HCH, heptachlor, aldrin, gamma chlordane, alpha-endosulfan, DDE, endrin, dieldrin, DDD, DDT, endosulfan sulfate, methoxychlor, ethoprophos, diazinon, dimethoate, pirimiphos-methyl, fenitrothion, malathion, chlorfenvinphos, profenofos, allethrin, bifenthrin, λ-cyhalothrin, permethrin, cyfluthrin, cypermethrin, fenvalerate, deltamethrin and chlorpyrifos. Supplementary information 5 (SI 5) provides the methods for the sample extraction and pesticides analysis.

#### Data analysis

Multivariate analyses were performed using the CANOCO 5 program to investigate the correlations among physicochemical characteristics of the sampling sites, the macroinvertebrate species and their relationships (Van den Brink et al., 2003; Ter Braak and Šmilauer 2018). For both the physicochemical and the macroinvertebrate data set the significance of the differences between the dry and the wet season was evaluated using an RDA analysis including season as explanatory variables and sampling date as covariables. Within the Monte Carlo permutation test following the RDA analyses, the samples were only permuted within the covariables. The significance of the differences between sampling times was tested using season as covariables and permuting the samples only within the covariables. After that, a PCA was performed for both data sets including season and sites as passive explanatory variables

Redundancy analysis (RDA) was used to test the significance of each the physicochemical parameters, as well as the substrate composition (Table S1) in explaining the differences in community composition. It was again used to examine the relationships between environmental variables (i.e. physicochemical and habitat parameters) and the abundance of macroinvertebrates. This analysis was followed by another RDA including the significant physicochemical and habitat parameters as explanatory variables and; season and sampling site as passive explanatory variables. The abundance values of macroinvertebrates were log (2x + 1) transformed in the above multivariate analysis, where x represents the abundance data (Van den Brink et al. 2000).

## Results and Discussions

### Physicochemical Parameters

Habitat assessments during the study were highly variable in the form of watershed features, riparian vegetation, in-stream features and substratum. Lower availability of the hard habitat like cobble substratum occurred at stations (Sedorm 1) T1, (WRI) T4, (ATL) T5, (Adi Lake Ref.) T6, (Kpong) T7 and (Akuse Sand Winning) T10. These stations had sand content ranging from 15 to 100% (Table S1).

**Fig. 2:**
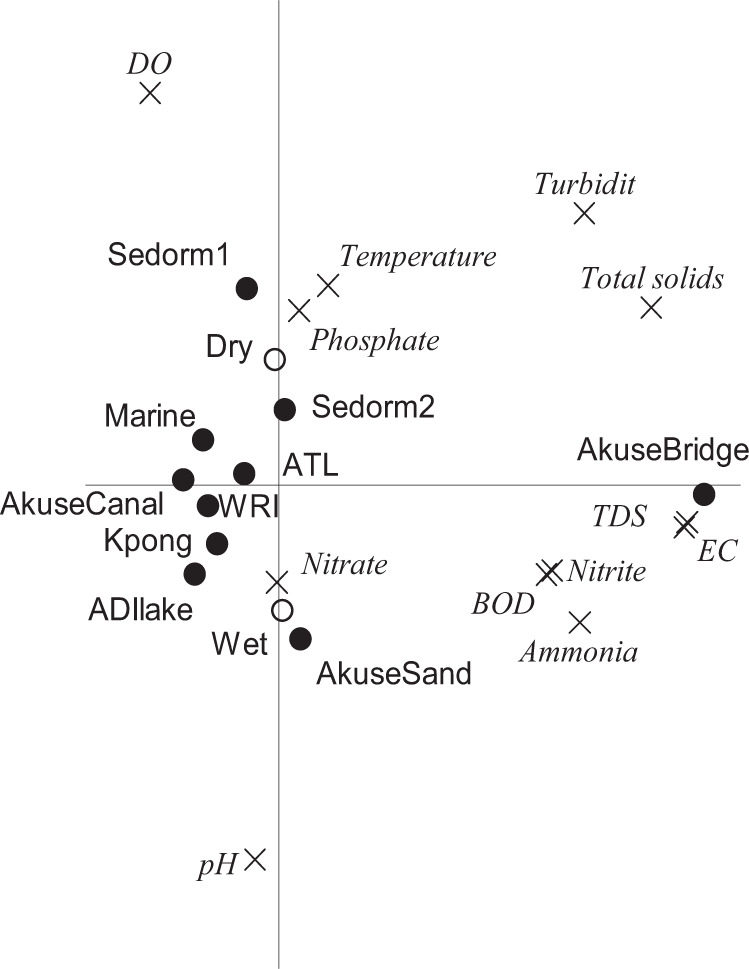
PCA plot showing the correlations between physicochemical parameters and their relative values in the different sites and seasons. The horizontal and vertical axes display 34 and 19% of the variation in physicochemical parameter values, respectively. Monte Carlo permutation tests indicated that differences between seasons and sites are significant (*p* = 0.002), while the differences between sampling dates was not significant (see text for test conditions and abbreviations)

There was a clear separation between physicochemical parameters and their relative values in the different sites and seasons in the PCA ordination diagram (Fig. 2). Additionally, there was a significant difference between seasons and sites (Monte Carlo permutation test; *p* = 0.002) while no significant difference existed between sampling dates (*p* > 0.05. This is in contrast to the assertion by Gampson et al. (2014) that physicochemical parameters do not vary much in terms of the sampling sites of the Lower Volta basin. Thus, the anthropogenic activities resulting from the adjoining land use characteristics, may have changed the physicochemical parameters. Again, the rainy season is characterized by a lot of precipitation which can influence the physicochemical parameters of the river. The PCA plot shows the largest differences in values between the stations for TDS, electrical conductivity (EC), turbidity, total solids, ammonia, pH ad DO (Fig. 2). Akuse Bridge is clustered away from all other stations, with relatively high TDS and EC values. The vertical axis merely displays the differences between the seasons, which were significant. The dry season recorded lower pH values compared to the wet season (Table SI 2; Fig. 2). The lowest pH of 4.4 was recorded at the first sampling of Sedorm 1 during the dry season which could be described as acidic (Table SI 2). The highest (10.25) were also recorded at Sedorm 1 and Marine but during the wet season. All the pH values determined in the wet season were within the WHO recommended range for drinking water (6.5–9.5) (Table SI 2) except the first and third sampling of Sedorm 1 and the third sampling of Marine in the wet season. This could be due to photosynthetic activity and microbial respiration as well as decomposing activities at the large expanse of wetland associated with Sedorm 1 thus affecting the pH value. Similar values have been reported on the Volta River by other studies (Gampson et al. 2014; Amoah and Koranteng 2006). Overall water temperature ranged from 28.1 to 32.8 °C (ATL, Sedorm) and 28.5 to 31.5 °C (Sedorm 2, Sedorm 1) in the dry and wet seasons, respectively (Table SI 2). The temperatures of the sampling sites were relatively constant and compares to the range (27–30 °C) reported by Amoah and Koranteng (2006).

Conductivity of the water samples ranged from 66 to 149 μS/cm (Akuse Canal, Akuse Bridge) and 68 to 166 μS/cm (WRI, Akuse Bridge) (Table SI 2) in the dry and wet seasons, respectively. The mean values obtained for both seasons were below the WHO recommended guideline limit of 1400 μS/cm. Conductivity is related to the concentration of TDS. The TDS values obtained for both the dry and wet season were below recommended limit of 500–1000 (mg/L) permissible for drinking (Davis, Dewiest (1966)). The electrical conductivity and TDS values obtained here indicates relatively low salt contents in the study area. The mean total solids of the water in the study area ranged from 42–99 mg/L in the dry season and 44–106 mg/L in the wet season, indicating good water quality. Turbidity values were comparatively higher in the dry season and ranged from 23 to 90 NTU, whiles the wet season recorded values of 3–26 NTU (Tables SI 2). Except Adi, Kpong and Akuse canal in the wet season (Table SI 2), all the samples in both seasons had turbidity values exceeding 5 NTU, the WHO guideline value for turbidity in drinking water (WHO World Health Organization (2004); WHO World Health Organization (2008)). The high turbidity may be attributed to the larger particles such as organic matter, dissolved solids, agricultural runoff, leaching of soil contaminant and point source water pollution discharged from industrial or sewage treatment plants. This causes problems with water purification processes, leading to increased treatment cost (DWAF Department of Water Affairs and Forestry (1998)).

Dissolved oxygen (DO) varied from 4.4–14.7 mg/L to 2.1–9.8 mg/L in the dry season and wet seasons, respectively (Table SI 2). The highest value was measured during the dry season at sampling site Marine and the lowest value was measured at site Akuse Sand Winning in the wet season. The low DO at some sites may be caused by the decomposition of organic matter, dissolved gases, industrial waste, mineral waste and landfill leachate (Table SI 1). Acceptable range of BOD concentrations for drinking water of 0.8–5 mg/L is set by WHO World Health Organization (2004); WHO World Health Organization (2008), but our study revealed ranges of 2.18–5.82 mg/L and 1.02–18.7 mg/L in the dry season and wet season, respectively (Table SI 2). The highest BOD value was recorded at the sampling site Akuse Sand Winning during the wet season (Table SI 2). The high levels obtained could possibly be attributed to domestic discharges which can increase the organic loads in the water (Table SI 1) a view shared by other researchers (Avalon Global Research, 2012; Edokpayi et al. 2017).

### Nutrients

The WHO has adopted a standard of 50 mg/L for nitrate-nitrogen and 3 mg/L for nitrite-nitrogen as the maximum contaminant level for drinking water (WHO World Health Organization (2004); WHO World Health Organization (2008)). Nitrate levels ranged between 0.1–1.7 mg/L in the dry season and 1.1–7.9 mg/L in the wet season. The ranges of nitrite were 0.01–0.03 mg/L (dry) and 0.01–0.05 mg/L (wet) and of ammonium were <0.001–0.65 mg/L (dry), 0.01–1.45 mg/L (wet), respectively (Table SI 2). These concentration levels were generally low and below the WHO standard. Criteria for total ammonia (NH_3_) have been established, for example by the EPA, to reflect the varying toxicity of NH_3_ with pH (USEPA United States Environmental Protection Agency (2013)). However, WHO does recognize odor effects at a concentration of 1.5 mg/L and taste effects at 35 mg/L. The highest NH_3_ concentration of 1.45 mg/L was recorded in this study for the wet season, so odor effects could occur, but not taste effects. In other studies, water quality criteria for phosphorus compounds, such as phosphates, are set at a concentration that prevents excessive growth of algae. Phosphorous is a limiting nutrient for algal growth and therefore controls the primary productivity of a water body (Karikari et al. 2007). It is also an essential nutrient and indicator of anthropogenic pollution. In most natural waters, PO4-P concentration range from 0.005 to 0.020 mg/L. In pristine waters, PO4-P concentrations may be as low as 0.001 mg/L (Karikari et al. 2007). Levels of PO4-P in this study varied between 0.16–4.97 mg/L in the dry season and 0.14–1.45 mg/L in the wet season. Digestive problems could occur in humans from drinking water with extremely high levels of phosphate (Morrison et al. 2001). None of the samples had values that exceeded the 5 mg/L set as standard in South Africa (Morrison et al. 2001).

### Pesticides

The concentration of organochlorine pesticides were below the detection limit (0.005 mg/L) at all the sampling sites. Meanwhile, Ntow (2005) reported gamma-HCH levels of 8 µg/L as well as alpha-endosulfan and endosulfan sulfate concentrations of 36 and 23 µg/L respectively in the Volta Lake. Settling of agricultural chemicals along with sediment could explain the low water concentrations. A study by Logah et al. (2017) suggests that there is high sediment concentrations downstream of Akuse which can be attributed to sand mining activities at various sections of the river and sediment input from tributaries could explain the low concentrations of agricultural pesticides in the water. Also, the absence of detection of organochlorines could be due to the ban of the use of e.g. DDT (GEPA Ghana Environmental Protection Agency (2008)) in Ghana, over time leading to possible degradation and dilution in the water body. Recent use of such products may also have been stopped which would have lowered the organochlorine pesticide levels. However, λ-cyhalothrin was detected at Sedorm 1 and Akuse Canal in the dry season in concentrations of 0.6 and 8.8 µg/L respectively. Cypermethrin was detected at a concentration of 1.4 µg/L at Marine during the January–February dry season sampling period. λ-cyhalothrin is highly lipophilic and tends to bind rapidly and strongly to organic materials (Maund et al. 1998; Leistra et al. 2003). Furthermore λ-cyhalothrin is highly toxic to some groups of aquatic organisms, particularly insects and crustaceans, with the midge *Chaoborus obscuripes* being sensitive (48- and 96-h EC_50_ = 0.0028 µg/L. Other insect larvae (Hemiptera, Ephemeroptera) and macrocrustacea (Amphipoda, Isopoda) are also relatively sensitive, with 48-and 96-h EC_50_ values between 0.01 and 0.1 µg/L (Schroer et al. 2004). Likewise, fish is sensitive with reported LC_50_ in bluegill sunfish, 0.21 μg/L; rainbow trout, 0.24 µg/L (Kidd and James 1991). Cypermethrin likewise is very highly toxic to fish and aquatic invertebrates. The LC_50_ (96-h) for cypermethrin and rainbow trout is 8.2 µg /L, and for bluegill sunfish is 1.8 µg/L while the effect concentrations for the total crustacean community and cladoceran and copepod subgroups in a study by Friberg-Jensen et al. (2003) ranged between 0.02–0.07 and 0.04–0.17 µg/L, respectively, with copepods being less sensitive than cladocerans. This raises concern as based on intrinsic sensitivity, biological traits, mode of action used for invertebrate vulnerability index rankings by Rico and Van den Brink (2015), Ephemeroptera, Plecoptera, Tricoptera, and Odonata genera were identified potentially most vulnerable to pyrethroids in aquatic ecosystems. The pesticide data were however not analysed further due to the low number of detections (Table SI 4).

### Macroinvertebrate Community

A total of 14 and 16 macroinvertebrate fauna were identified in the dry and wet seasons, respectively, belonging to 2 major phyla viz: Arthropoda and Mollusca. Among these phyla, Arthropoda (*Polypedilum fuscipenne, Stereo chironomus* sp. *Ictinogamphus* sp. *Laccophilus* sp. *Centroptiloides* sp. *Hagenius* sp. *Lethocerus* sp. *Phaon Iridipennis*, *Centroptiloides, Culicidae* sp. and *Eurymetra* sp.) dominated (66.7%) followed by Mollusca (*Physa* sp. *Lymnaea glabra*, *Mya arenaria, Bithynia* sp. and *Pomacea paludosa*) (33.3%) (Table SI 3).). It should be noted that three taxa (*Bithynia* sp., *Eurymetra* sp. and *P. paludosa*) were only found at one sampling site, so their results should be interpreted with caution. Generally, there was low species richness and presence of tolerant taxa and change in hydrology. Hydropeaking leading to rise and fall of water levels could cause desiccation of several invertebrate taxa, see, for example Abernethy et al. (2021), as well as considerable change in geomorphology of the river, particularly as a result of sustained high releases from the Akosombo and Kpong dams (Logah et al. 2017).

**Fig. 3:**
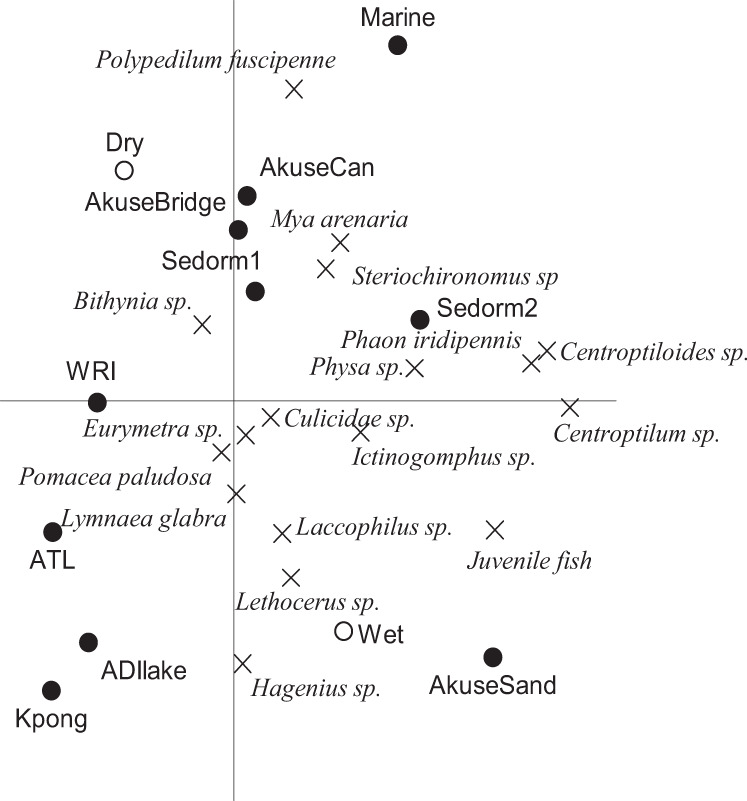
PCA plot showing the correlations between macroinvertebrate abundance values in the different sites and seasons. The horizontal and vertical axes display 27 and 16% of the variation in the abundance of macroinvertebrate species, respectively. Monte Carlo permutation tests (999 permutations) indicated that differences between seasons and sites are significant (*p* = 0.002), while the differences between sampling dates was not significant (see text for test conditions)

Significant difference existed between macroinvertebrate composition at the different sampling sites and seasons (Monte Carlo permutation tests; *p* = 0.002; Fig. 3). The macroinvertebrates were generally more abundant in the wet season than the dry season except *P. fuscipenne* and *Bithynia* sp. at Akuse Canal, Akuse bridge and Marine (Fig. 3). The Akuse and Marine sites are, however, characterized by industrial, township and agricultural activities (Table SI 1). Similar results have been reported in the Porto-Novo lagoon in Benin (Adandedjan et al. 2011). *L. glabra* was the predominant macroinvertebrate in both seasons (Fig. 3) because it can survive under polluted and unpolluted conditions (Rondelaud et al. 2009). The species with high frequency included *L. glabra* (Lymnaeidae; Gastropoda), *P. fuscipenne* (Chironomidae; Diptera), *Centroptiloides* sp. (Baetidae; Ephemeroptera), *Physa* sp. (Physidae; Gastropoda) and *Steriochironomus* sp. (Chironomidae; Diptera) (Fig. 3; Table SI 3). At the sites where human pressures were present (anthropogenic stress, agricultural waste and domestic waste, i.e. Akuse and Sedorm sites) taxa tolerant to pollution, such as Chironomidae were abundant, as well as even some non-tolerant ones increased (e.g. some Ephemeroptera families). *Physa* sp. has been used as a pollution indicator in Australia by Shield et al. (2014) and has also been found abundant in the study areas (Akuse and Sedorm sites and WRI) where agricultural, aquaculture, waste, organic and sewage pollution is high. Also, in a study by Hynes, (1975a, b), Ephemeroptera (*Centroptiloides* sp. and *Centroptilum*) were mentioned as playing a major role in the recovery and recolonization of zoobenthos of a dried up river (Pawmpawm River, Southern Ghana), showing their high recolonization capacity.

### Correlation among the Physicochemical Parameters and Macroinvertebrates

**Fig. 4:**
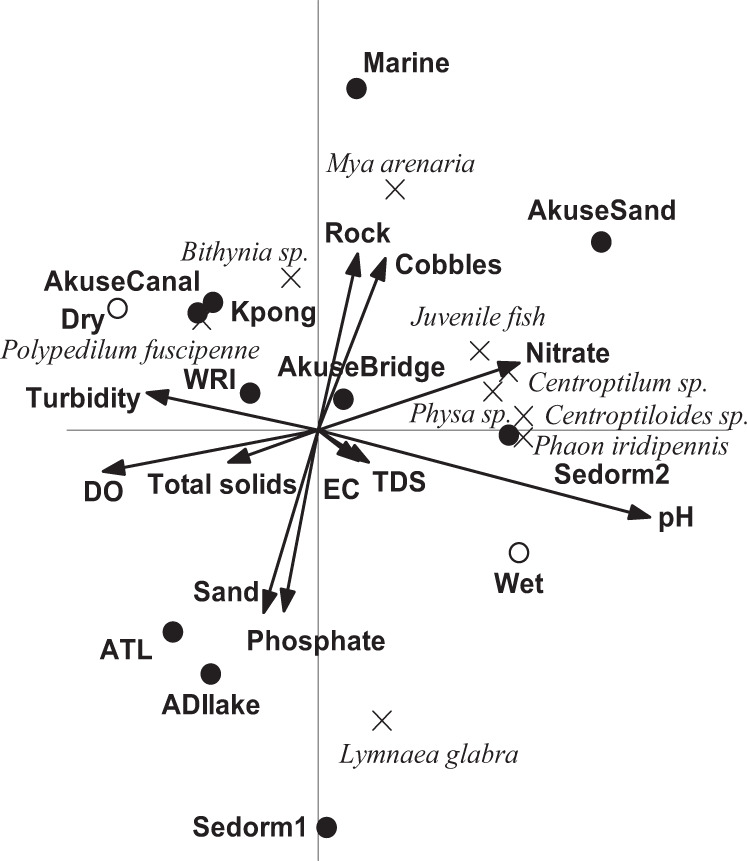
RDA biplot showing the environmental variables that significantly explained the variation in macroinvertebrate composition between stations as result of the Monte Carlo permutation tests (999 permutations; *p* < 0.10). The environmental variables explained 34% of the variation in species composition of which 35% is displayed on the horizontal axis and another 27% on the vertical axis. For clarity, only 9 out of 17 species are shown, these are the species which best fitted the ordination space (see text for abbreviations)

We found that pH, DO, TDS, turbidity, EC, nutrients and substratum together explained around 34% of the total variation in macroinvertebrate composition among sites (Fig. 4). Species on the left-hand side of the diagram, such as *P. fuscipenne* correlate significantly positively with turbidity and DO concentrations and occurred in relatively high abundance values at the Kpong and Akuse canal sampling sites during the dry season. Likewise, species on the right-hand side of the diagram, including *Physa* sp*. Centroptilum* sp*. Centroptiloides* sp*. Phaon iridipennis* and juvenile fish were positively correlated with nitrate concentration and pH. In contrast, these species also were negatively correlated with turbidity and DO, and occurred in higher abundance at Sedorm 2 and Akuse Sand sampling sites during the wet season (Fig. 4). Ishaq, KhanA (2014), observed an inverse correlation of macroinvertebrates with turbidity and a positive relationship with pH, confirming our results. The molluscs (*Mya arenaria and Bithynia* sp.) were negatively correlated with the sand substratum and phosphate concentration; however, *L. glabra* was positively correlated (Fig. 4). The results suggest that the nature of the substratum and organic contamination caused by anthropogenic activities might be a primary force in determining benthic community composition. For instance, absence of benthic macroinvertebrates was observed in samples from ATL where high levels of nutrients were determined (Tables S2 and S3). For instance, a study by Liston et al. (2008) suggests that, total macroinvertebrate density in benthic floc eventually decreases with enrichment. Macroinvertebrate density first increases with enrichment until periphyton mats are lost, after which it decreases due to a loss of habitat.

Overall, our results suggest that anthropogenic disturbance (i.e. environmental pollution) significantly contributed to the variation in benthic assemblages in rivers, even though we cannot rule out the influence of unmeasured ecological drivers.

## Conclusions

The results of this study show that macroinvertebrate community composition shifted along the physicochemical parameters, site and season. There were significant correlations between macroinvertebrate communities and environmental variables (i.e. DO, turbidity, substratum, total solids, EC, TDS, pH, and nutrients) in the Volta river. There was also a significant relationship between macroinvertebrate community composition and sampling sites. Absence of benthic macroinvertebrates was recorded at a few samples sites of the Volta river where high levels of nutrients were determined. Our results suggest that anthropogenic activities (e.g. aquaculture, agriculture effluent discharges) altered the macroinvertebrate community composition directly or indirectly in the exposed sampling sites.

## Supplementary Information

Supplementary Information
